# Some like it hot, but not too hot

**DOI:** 10.7554/eLife.12838

**Published:** 2015-12-15

**Authors:** Chloe Greppi, Gonzalo Budelli, Paul A Garrity

**Affiliations:** Department of Biology and National Center for Behavioral Genomics, Brandeis University, Waltham, United States; Department of Biology and National Center for Behavioral Genomics, Brandeis University, Waltham, United States; Department of Biology and National Center for Behavioral Genomics, Brandeis University, Waltham, United Statespgarrity@brandeis.edu

**Keywords:** *Aedes aegypti*, mosquito, behavior, Other

## Abstract

A temperature-sensitive receptor prevents mosquitoes from being attracted to targets that are hotter than a potential host.

**Related research article** Corfas RA, Vosshall LB. 2015. The cation channel TRPA1 tunes mosquito thermotaxis to host temperatures. *eLife* **4**:e11750. doi: 10.7554/eLife.11750

**Image** Mosquitoes find hosts by homing in on body heat (Image credit: Roman A Corfas and Benjamin J Matthews)

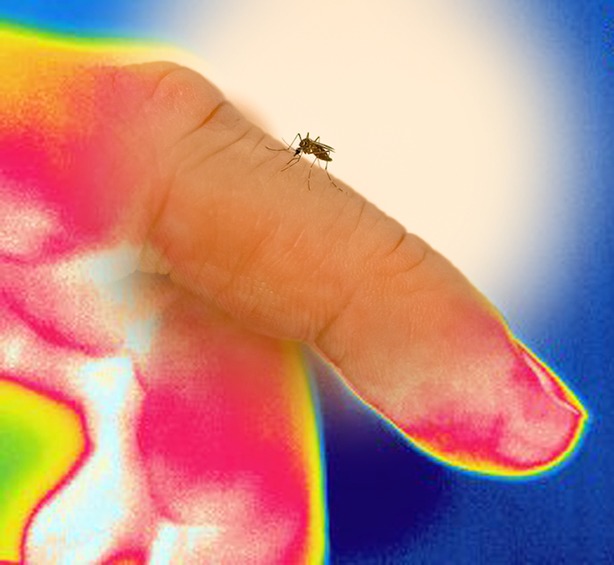

From warm summer days to cold winter nights, temperature is a ubiquitous sensory stimulus. All animals rely on their ability to detect environmental temperatures to avoid harm and to seek out more optimal conditions. Some animals, such as mosquitoes, also use their temperature sensors for a more nefarious purpose: to locate warm prey for a blood meal.

The ability of the mosquito to home in on warm bodies was first recognized over a century ago (Brown, 1951; Howlett, 1910), but the details of this behavior are still not fully understood. Now, in eLife, Roman Corfas and Leslie Vosshall from Rockefeller University report on the molecular basis of temperature-sensing behavior in *Aedes aegypti*, the mosquito that spreads yellow fever (Corfas and Vosshall, 2015). They show how avoiding high temperatures can stop these insects from being attracted to targets that are too hot to represent a suitable host: in other words, while these mosquitoes like it hot, they don’t like it *too* hot.

Corfas and Vosshall’s study is grounded in previous work on the fruit fly *Drosophila melanogaster* (Barbagallo and Garrity, 2015). Fruit flies use at least two molecular receptors to guide their movements in response to warmth (Hamada et al., 2008; Ni et al., 2013), and while about 250 million years of evolution separate flies and mosquitoes, versions of each receptor are present in *A. aegypti*. It is unclear precisely where these receptors, TRPA1 and GR19, are expressed in *Aedes* mosquitoes, but in the malaria-spreading mosquito *Anopheles gambiae*, TRPA1 is expressed at the tips of the antennae (Wang et al., 2009). This is intriguing, because the tip of a mosquito antenna houses very sensitive thermoreceptors that could help drive host-seeking behavior (Davis and Sokolove, 1975).

Female mosquitoes normally prefer temperatures around 23°C. However, a puff of carbon dioxide (which could indicate that a metabolically active host is nearby) drives the mosquitoes to seek out temperatures that are closer to the body temperature of a mammal or bird (that is, between about 37°C and 43°C; McMeniman et al., 2014). Corfas and Vosshall started by further characterizing this heat-seeking behavior. They found that mosquitoes were strongly attracted to a target when it was heated to temperatures above ambient, but only up to ~50°C. When it got hotter, this attraction declined strongly.

To probe the molecular mechanisms that might control this response, Corfas and Vosshall exploited genome-editing techniques to knock out the genes for GR19 and TRPA1 in *A. aegypti*. They found that mosquitoes lacking GR19 behaved like wild type mosquitoes and showed normal responses to heat. However, mosquitoes without TRPA1 continued to be attracted to the target even when its temperature reached potentially harmful levels (> 50°C).

While the ability of animals to avoid high temperatures is commonly viewed from the perspective of damage avoidance, Corfas and Voshall raise the possibility that this response could also help a heat-seeking mosquito to choose among multiple potential targets. In fact, when tested for their ability to discriminate between two hot targets (one at 40°C, the other at 50°C), TRPA1-knockout mosquitoes showed a greatly reduced preference for a 40°C target over a 50°C target. Thus, while TRPA1 is not required for heat seeking, per se, it helps to set an upper limit on the temperatures mosquitoes seek, which prevents them from being attracted to stimuli warmer than their hosts.

While the work of Corfas and Voshall advances our understanding of temperature sensing in *A. aegypti*, the identity of the receptor (or receptors) that drives heat-seeking behavior remains elusive. However, the recent use of rapid genome-editing techniques in *Aedes* mosquitoes (including the CRISPR-Cas9 system; Kistler et al., 2015), has dramatically simplified the study of the function of genes in this organism. This greatly increases the likelihood that more of the molecules behind heat seeking and other mosquito sensory responses will be uncovered in the near future. As mosquito-borne illnesses kill more than a million people every year, interventions that can reduce the spread of such diseases are crucial. It is hoped that an increased understanding of how mosquitoes target their hosts can help accelerate the development of new control strategies.

## References

[bib1] Barbagallo B, Garrity PA (2015). Temperature sensation in *Drosophila*. Current Opinion in Neurobiology.

[bib2] Brown AWA (1951). Factors in the attractiveness of bodies for mosquitoes. Nature.

[bib3] Corfas RA, Vosshall LB (2015). The cation channel TRPA1 tunes mosquito thermotaxis to host temperatures. eLife.

[bib4] Davis EE, Sokolove PG (1975). Temperature responses of antennal receptors of the mosquito, *Aedes aegypti*. Journal of Comparative Physiology.

[bib5] Hamada FN, Rosenzweig M, Kang K, Pulver SR, Ghezzi A, Jegla TJ, Garrity PA (2008). An internal thermal sensor controlling temperature preference in *Drosophila*. Nature.

[bib6] Howlett FM (1910). The influence of temperature upon the biting of mosquitoes. Parasitology.

[bib7] Kistler KE, Vosshall LB, Matthews BJ (2015). Genome engineering with CRISPR-Cas9 in the mosquito *Aedes aegypti*. Cell Reports.

[bib8] McMeniman CJ, Corfas RA, Matthews BJ, Ritchie SA, Vosshall LB (2014). Multimodal integration of carbon dioxide and other sensory cues drives mosquito attraction to humans. Cell.

[bib9] Ni L, Bronk P, Chang EC, Lowell AM, Flam JO, Panzano VC, Theobald DL, Griffith LC, Garrity PA (2013). A gustatory receptor paralogue controls rapid warmth avoidance in *Drosophila*. Nature.

[bib10] Wang G, Qiu YT, Lu T, Kwon H-W, Jason Pitts R, Van Loon JJA, Takken W, Zwiebel LJ (2009). *Anopheles gambiae* TRPA1 is a heat-activated channel expressed in thermosensitive sensilla of female antennae. European Journal of Neuroscience.

